# Antimicrobial resistance in *Klebsiella pneumoniae*: identification of bacterial DNA adenine methyltransferase as a novel drug target from hypothetical proteins using subtractive genomics

**DOI:** 10.5808/gi.22067

**Published:** 2022-12-30

**Authors:** Umairah Natasya Mohd Omeershffudin, Suresh Kumar

**Affiliations:** 1 Post Graduate Centre, Management and Science University, Shah Alam 40100, Malaysia; 2 Faculty of Health and Life Sciences, Management and Science University, Shah Alam 40100, Malaysia

**Keywords:** anti-bacterial agents, DNA methylation, drug design, genomics, *Klebsiella pneumoniae*, protein domains

## Abstract

*Klebsiella pneumoniae* is a gram-negative bacterium that is known for causing infection in nosocomial settings. As reported by the World Health Organization, carbapenem-resistant Enterobacteriaceae, a category that includes *K. pneumoniae*, are classified as an urgent threat, and the greatest concern is that these bacterial pathogens may acquire genetic traits that make them resistant towards antibiotics. The last class of antibiotics, carbapenems, are not able to combat these bacterial pathogens, allowing them to clonally expand antibiotic-resistant strains. Most antibiotics target essential pathways of bacterial cells; however, these targets are no longer susceptible to antibiotics. Hence, in our study, we focused on a hypothetical protein in *K. pneumoniae* that contains a DNA methylation protein domain, suggesting a new potential site as a drug target. DNA methylation regulates the attenuation of bacterial virulence. We integrated computational-aided drug design by using a bioinformatics approach to perform subtractive genomics, virtual screening, and fingerprint similarity search. We identified a new potential drug, koenimbine, which could be a novel antibiotic.

## Introduction

*Klebsiella pneumoniae* belongs to the family of *Enterobactericeae* and is classified as an example of carbapenem-resistant Enterobacteriaceae. This organism causes infections in nosocomial settings, posing a global threat due to the ability of bacterial pathogens to acquire mobile genetic traits, making them resistant towards antibiotics. *K. pneumoniae* causes a wide range of infections, including urinary tract infections, pneumonia, and liver abscesses [1]. Current drugs target the cellular processes of bacterial pathogens, such as translation, transcription, and replication. However, bacterial pathogens can still develop resistance to antibiotics [2].

The emergence of multidrug-resistant (MDR) bacterial pathogens has become a global threat, as stated by the World Health Organization [3]. These pathogens can acquire genetic traits that allow them to develop resistance, leading to an increase in prevalence and affecting human populations by lowering mortality and morbidity rates. Current antibiotics are unable to effectively combat MDR pathogens, which can acquire mobile genetic traits that make them resistant to antibiotics [4]. Carbapenems and colistin are among the most potent antibiotics, but some bacteria, such as *K. pneumoniae* carbapenemase-producing strains have developed resistance to them [5,6].

DNA adenine methyltransferase (Dam) is a promising drug target because it plays a role in the epigenetic regulatory machinery that helps sustain the viability of bacterial pathogens and regulates their pathogenicity [7]. DNA methylation is an epigenetic mechanism that regulates various bacterial physiological processes, such as chromosome replication, DNA segregation, mismatch repair, transposition, and transcription, by altering the affinity and interaction of regulatory proteins with DNA. Dam, which methylates the N-6 position of adenine in the GATC sequence, is essential for activating bacterial virulence genes. Research on antibiotic resistance has increasingly linked Dam, a DNA inhibitor that plays a critical role in bacterial pathogenesis, to antibiotic resistance. Dam is required for the replication and gene expression of the bacterium. The finding of Dam in epigenetics studies makes it easier to discover medications for this MDR pathogen. Dam modification is also important in bacterial pathogenicity, as pathogenesis is influenced by either deficiency or overexpression, which is believed to induce attenuation, or premature transcription in the bacterium. Since most drug development focuses on virulence factors rather than mechanisms that maintain the viability of pathogenic bacteria, Dam systems can be targeted as potential antibiotic targets. The underlying mechanism of Dam's biological role makes it an appealing target for antibiotics. In this study, we used a hypothetical protein from *K. pneumoniae* that contains a Dam domain, which has been linked to antibiotic resistance [8]. In this study, we used a hypothetical protein from *K. pneumoniae* that contains a Dam domain, which has been linked to antibiotic resistance. We employed bioinformatics techniques, including subtractive genomics, virtual screening, and fingerprint similarity searches, to aid in computer-aided drug discovery. We identified a new potential drug, koenimbine, which can be further explored for drug development processes.

## Methods

**Fig. 1.**
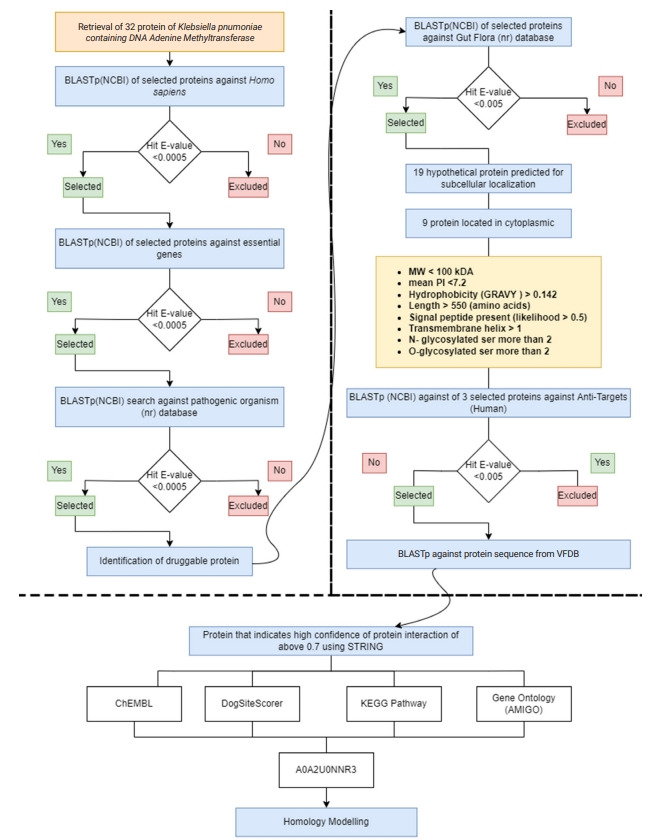
Overall workflow of the identification of proteins as putative drug targets. KEGG, Kyoto Encyclopedia of Genes and Genomes; STRING, Search Tool for the Retrieval of Interacting Genes/Proteins; VFDB, Virulence Factor Database.

This study involved several *in silico* methods, including subtractive genomic analysis, molecular docking, and absorption, distribution, metabolism, excretion, and toxicity (ADMET) testing. The workflow is summarized in Fig. 1.

### Retrieval of the bacterial proteome

The UniProt database was used to obtain all available *K. pneumoniae* hypothetical protein sequences containing Dam by using the keyword "*Klebsiella pneumoniae*" and the term "n6 DNA adenine methyltransferase." The UniProt database is the largest protein database with detailed annotations of proteins [9].

### Identification and analysis of non-homologous sequences

All retrieved hypothetical protein sequences were screened to select only non-homologous sequences. This was done using BLASTp (Basic Local Alignment for Protein) against the National Center for Biotechnology Institute database (NCBI) with a threshold of an e-value of <0.0005 [10].

### Identification of essential genes

To identify a potential drug target, the non-homologous hypothetical protein must contain essential genes that are important for the cellular processes of the cell. These essential genes are important because they are involved in major constituents of the cells necessary for the survival of the pathogens. The hypothetical proteins were screened using BLAST against the Database of Essential Genes (DEG) with a threshold of an e-value of <0.0001 [11].

### Broad-spectrum analysis

To be considered a broad-spectrum hypothetical protein, a protein must be present in more than 25 bacterial protein kingdoms. Hypothetical proteins with essential genes were screened using BLASTp, with an e-value of 0.005.

### Druggability analysis

DrugBank is a comprehensive database used for *in silico* computational-aided drug design that includes information on drug targets and the actions of drugs that have been approved by the Food and Drug Administration (FDA) [12]. A hypothetical protein was screened using BLASTp against the DrugBank database with an e-value of 0.001 to determine if it was a druggable protein.

### Non-homology analysis against gut microbiota

Gut microbiota plays an important role in the human gastrointestinal. A homologous protein with similarity to the human gut may interact and bind with the gut flora proteins, leading to adverse pharmacokinetic side effects in the host. Hence, any homologous protein that was similar to the human gun was removed by using BLASTp with an e-value of 0.0001 [13].

### Subcellular localization

The subcellular localization of the hypothetical protein was determined using PSORTb 3.0, an accurate predictor of bacterial protein subcellular localization. Gram-negative bacterial proteins have five major localizations: cytoplasmic, inner membrane, periplasmic, outer membrane, and extracellular [14]. In this study, proteins located at the membrane channel and cytoplasmic were selected because they are more likely to be good drug targets [15]. Based on the subcellular localization identification using PSORTb, and drug targets located in the cytoplasm were selected.

### Drug target property

Drugs often target enzymes and are involved in binding, signaling, and communication. According to Bakheet [16], good drug targets have eight key properties: hydrophobicity >–142.4, amino acid length >550, presence of a signal motif, absence of a PEST motif, more than two N-glycosylated amino acids, no more than one O-glycosylated serine, mean pI < 7.2, and presence of a transmembrane helix with a cytoplasmic membrane location.

To analyze these properties, the ExPasy server was used to determine amino acid length, hydrophobicity, and pI [17]. The presence of a transmembrane helix (THMM), was identified using the TMHMM method (http://www.cbs.dtu.dk/∼krogh/TMHMM/) [18], and PEST regions were identified using the Epestfind tool (http://emboss.cbr.nrc.ca/cgi-bin/emboss/epestfind). To analyze O-glycosylation, the NetOglyc program (http://www.cbs.dtu.dk/services/NetOGlyc/) was used, while N-glycosylation was evaluated using a specialized tool (http://www.cbs.dtu.dk/services/NetNGlyc/) [19].

### Anti-target non-homology analysis

Anti-target non-homology analysis was performed to eliminate anti-target receptors [20], using an e-value of 0.005.

### Drug data properties

The ChEMBL database provides bioactivity, molecule, target, and drug data from various sources, including medicinal chemistry literature, and can be used to identify good drug targets [21]. Hypothetical proteins that showed more matches from the ChEMBL were considered to be good drug targets.

### Virulence factor analysis

The Virulence Factor Database (VFDB) provides an extensive understanding of the virulence factors characterized by 16 dominant bacterial pathogens [22]. These virulence factors are crucial in causing bacterial pathogens to colonize the host and harm the host cell.

### Protein-protein interactions

Protein functions are a key component of the cellular phenotype and are not independent. Networks of interacting proteins help to understand protein function. To obtain protein-protein interactions of *K. pneumoniae*, the Search Tool for the Retrieval of Interacting Genes/Proteins (STRING) database was used [23]. Neighboring proteins with a high confidence score (greater than 0.7) were included.

### Binding site prediction

Drugs bind to specific sites on proteins. The interactions between these binding sites help to understand the physicochemical interactions between drugs and proteins. These predictions were made using DoGSiteScorer, an automated algorithm for pocket and drug ability prediction. Pockets were predicted by mapping the protein to a grid and using the Gaussian difference to filter and identify pocket regions on the protein surface [24].

### Metabolic pathway analysis

Comparative metabolic pathway analysis was performed to identify unique interactions between the host and the identified protein using the Kyoto Encyclopedia of Genes and Genomes (KEGG). The output provides KEGG Orthology assignments that generate KEGG pathways [25]. A metabolic pathway analysis is essential to elucidate the predicted putative drugs.

### Gene ontology

As the identified protein is uncharacterized, it is important to identify its specific biological role. Understanding the biological role of a protein provides insight into its specific function [26]. The biological role of the identified protein was assessed via Gene Ontology (GO). a consortium for biology unification in shared eukaryotes that constructs three ontological categories: biological processes, molecular functions, and cellular components [27]. The GO classification provides essential information on biological role of proteins in specific organisms.

### Homology modeling

The structures of uncharacterized proteins are not available in the Protein Data Bank (PDB), although structural mechanism is vital for an understanding of ligand interactions and channel interactions with the targeted protein [28]. The targeted 3D protein structure was constructed by using the fully automated server SWISS-MODEL. First, homology modeling of the targeted protein was compared against a similar protein structure template [29]. The template for the identified protein was identified based on the protein structure of *Escherichia coli* bacteria containing Dam (PDB ID: 4RTL) [30]. The modeled protein structure was then verified to check the protein quality of the stereochemical structure using PROCHECK [31], ProSA-web where errors in the 3D structure are recognized [32], and ERRAT.

### Ligand preparation

Seven ligands were identified as DNA methyltransferase inhibitors from a literature review [33]: mahanine (PubChem ID: 375151), curcumin (PubChem ID: 969516), epigallocatechin gallate (PubChem ID: 65064), nanaomycin A (PubChem ID: 40586), parthenolide (PubChem ID: 7251185), quercetin (PubChem ID: 5280343), and trimethylaurintricarboxylic acid (PubChem ID: 263071). Ligands developed for putative drug targets should not violate the five Lipinski rules; therefore, they were first validated using the SWISS-ADME server. Ligands that resulted in any violation were not further included in the analysis.

The 2D structure was obtained in SDF format and retrieved from the PubChem database (https://pubchem.ncbi.nlm.nih.gov/). The SDF file was converted to PDB via OpenBABEL and SMILES (http://cactus.nci.nih.gov/services/translate/) [34]. The converted PDB structures were minimized to PDBQT with the AutoDock Vina tool. Non-polar hydrogens were added and merged to the ligands, and Gasteiger charges were computed. The torsion of the ligand was defined and saved as a PDBQT extension.

### Molecular docking

Molecular docking is performed to identify ligands that bind with the lowest affinity score to develop potential putative drug targets by using AutoDock Vina. The default exhaustiveness was set to 1.0 Å. An identified protein was configured by first adding all hydrogens to the protein and merging the non-polar hydrogens. The Gasteiger charges were computed and the protein was saved as a PDBQT file.

A grid box was set based on the predicted binding site with the configuration values of the center grid box of x, y, z. The size of the dimension grid box was set to 30.0 Å. The binding affinity score was observed.

### Identification of novel inhibitors through a molecular fingerprint search of the prioritized ligand

To identify novel inhibitors, a fingerprint search was performed using NPASS (Natural Product Activity and Species Source databases) to search for compounds similar to the prioritized ligand based on the docking of DNA methyltransferase inhibitors [35]. The fingerprint search was done using by inputting the SMILES of prioritized ligand and setting the fingerprint type (pubchem-881 fp) with a threshold ≥ 0.90 in the search by structure and function in the NPASS database.

### Virtual screening

Virtual screening was performed to evaluate docking against clusters of ligands by using AutoDock. The settings were the default parameters of 1.0 Å with a dimension grid of 30.0 Å. The analysis was performed based on the binding energy score.

### ADMET testing

ADMET are the major processes carried out by the body as soon as a drug is administered [36]. These pharmacokinetic properties were evaluated to indicate the site of action of a drug using the pkCSM database [37]. The pkCSM database optimizes these pharmacokinetics properties by using the graph-based signatures.

## Results and Discussion

**Fig. 2.**
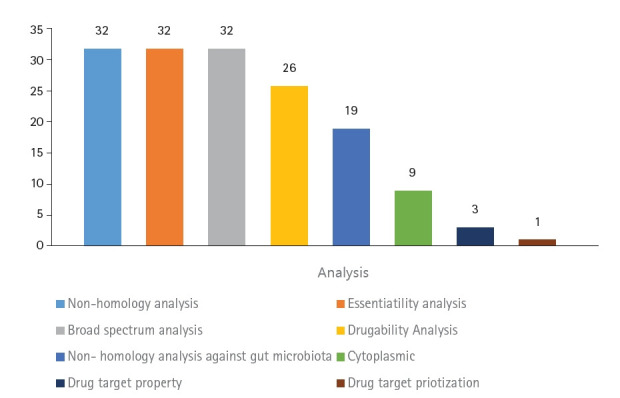
Summary of the subtractive genomic analysis of the 32 hypothetical proteins of *Klebsiella pneumoniae* containing DNA adenine methyltransferase.

The aim of this study was to identify novel DNA methyltransferase inhibitors for the bacterial species *K. pneumoniae*. All 32 hypothetical proteomes of *K. pneumoniae* containing Dam were retrieved from UniProt and analyzed as potential druggable proteins. The proteins were characterized using a subtractive genomics approach based on the following criteria: non-homology to the human host, presence of essential genes, broad-spectrum presence in the bacterial kingdom, and non-homology to the human gut microbiota. The workflow and analysis summary can be found in Figs. 1 and 2.

The first step of the subtractive genomics approach was non-homology analysis. Homologous proteins present in the human host may interact with molecules and carry unwanted toxicity. To decrease the risk of adverse side effects, non-homologous proteins were selected as putative drug targets by subjecting the protein sequences to BLAST with an e-value of 10^-3^. Essential genes are known to be essential for the survival of bacterial proteomes by maintaining cellular processes [11]. To identify essential genes in the bacterial proteomes, the sequences were subjected to BLASTp against the DEG database with an expected value of <0.0001.

To effectively treat multiple bacterial infections, the target drug-able protein should be common in the broad-spectrum bacterial kingdom [38]. Multiple target antibacterials are preferred as drugs. To predict whether these bacterial proteomes are broad-spectrum, all 32 proteins were searched using BLAST with an e-value of <0.0001 against the NCBI bacterial pathogens database. This resulted in all 32 proteins being non-homologous, containing essential genes, and present in a broad spectrum of the bacterial kingdom.

### Druggability analysis

Proteins that are druggable are defined as being able to bind strongly with drug molecules [39]. These are known as high-affinity bindings between the protein and ligand, which result in stronger intermolecular forces. A notable source of comprehensive drug data is DrugBank, which contains small molecule drugs, biotech drugs approved by the FDA, nutraceuticals, and experimental drug entries [12]. The targeted proteins were then searched against the DrugBank database using BLASTp, and of the 32 proteins, only 26 were characterized as druggable.

### Human gut microbiota analysis

The gut microbiota refers to the large population of organisms that colonize the intestinal tracts [40]. The gut microbiota is highly associated with human inflammatory diseases. Pathogens in the human gut microbiota co-evolved through a symbiotic relationship, promoting the replication of pathogens [41]. Homologous proteins may lead to unintentional blockage of proteins in the gut flora, causing adverse effects [42]. To prevent this, homologous proteins were removed by searching against the NCBI database of gut flora using BLASTp with a threshold of <0.005. Of the 26 proteins, 19 were found to be non-homologous to the human gut.

### Subcellular localization analysis

The characterization of subcellular localization is an important determinant in the development of putative drug targets, as it reveals the main function of the protein [43]. The localization of a protein determines its function [44]. Proteins located in cytoplasmic regions are more favorable as drug targets because they contain an abundance of enzymes, making them more feasible as drug targets. The cellular localization was predicted using PSORTb. Nine proteins were found to be located at the cytoplasmic membrane, while the localization of 10 proteins was unknown.

### Drug target property analysis

To further understand the drug properties of these nine proteins, they were analyzed based on eight key properties summarized by Bakheet and Doig [16]. These properties, which are important for good drug targets, include: hydrophobicity >–142.4, amino acid length >550, presence of a signal motif, absence of a PEST motif, more than 2 N-glycosylated amino acids, not more than 1 O-glycosylated serine, mean pI <7.2, presence of a transmembrane helix, and cytoplasmic membrane location.

One key aspect of good drug targets is high hydrophobicity. The balance of hydrophobicity in a protein is important for its folding and aggregation [45]. The higher the hydrophobicity of the protein, the better the folding, which indirectly affects its function [44]. The stabilization of hydrophobicity can also affect the binding affinity between the protein and ligand [46]. The results showed that all of the proteins had a hydrophobicity of <–0.142.

The isoelectric point (pI) of a protein, which reflects the overall charge of its amino acids, is another important factor to consider. The pI value determines the pH of the protein and its solubility. Higher pI values indicate that the protein is basic, while lower values indicate that it is acidic. A good drug target should have a pI value < 7.2. Three proteins were identified as having a mean pI < 7.2: A0A3P4EC49, A0A3P4UG76, and A0A2U0NNR3 (Table 1).

The desired amino acid length for a drug target should be greater than 550 amino acids in total length. The longer the amino acid length, the greater the surface area of the protein for interactions with drugs. However, all of the drug targets had less than 550 overall amino acids, which may be due to the type of bacteria species. Signal peptide cleavage aids in the transportation of proteins of the endoplasmic reticulum across the membrane [47]. However, the results indicated that the presence of a signal peptide itself is less significant due to the localization of the protein in the cytoplasm.

PEST regions are regions of a peptide that are rich in proline (P), glutamic acid (E), serine (S), and threonine (T). Proteins that have one or more PEST regions are associated with shorter intracellular half-lives, as they are reported to cause protein degradation [48]. All of the protein sequences observed contained at least one PEST region. However, transmembrane helices, which are amino acids that flank regions, were absent from all observed proteins.

Glycosylation is a crucial process that occurs abundantly in polypeptide chain modifications [49]. Bacterial proteins possess two glycosylation states: N-linked and O-linked glycosylation [50]. According to Bakheet [16], most bacterial protein drug targets either have more than two N-glycosylated amino acids or one or no O-glycosylated ser. Four proteins had one or no O-linked glycosylated serines, and three proteins had more than two N-linked glycosylated amino acids.

Based on these drug properties, three proteins were selected for further screening as they possessed more drug target characteristics: A0A3P4EC49, A0A3P4UG76, and A0A2U0NNR3 (Table 1).

### Identification of putative drug targets

It is necessary to predict which drug candidates are likely to fail. Therefore, a protein needs to first be identified as either an anti-target or target protein. An anti-target protein is a protein receptor that causes adverse pharmacokinetic side effects when it binds to the drug. Here, none of the three proteins were anti-target proteins.

Out of these three proteins, A0A2U0NNR3 had more desirable drug properties when analyzed using chEMBL, with 10 matches. It also showed the highest pocket binding score of 0.82 when analyzed using DoGSiteScorer, The protein was then further studied for its metabolic pathways. Based on the metabolic pathway analysis on the KEGG server, the protein was found to be involved in a unique pathway of DNA mismatch repair. The GO analysis describing the specific biological role of the protein indicated that the protein functions as a site-specific DNA methyltransferase (adenine-specific activity).

In order to understand the virulence mechanism, the proteins were queried in the VFDB. The bacterial protein virulence factors were as follows: VFG010749 (sdhB) Dot/Icm type IV secretion system effector, VFG001959 (hddC) capsular polysaccharide heptosyltransferase, VFG000166 (pchE) dihydroaeruginoic acid synthetase PchE [Py], VFG000272 (ureE) urease accessory protein (ureE), metalloch, and VFG002139 (cdsD) type III secretion system in the inner membrane (Table 2).

### Protein-protein interactions

**Fig. 3.**
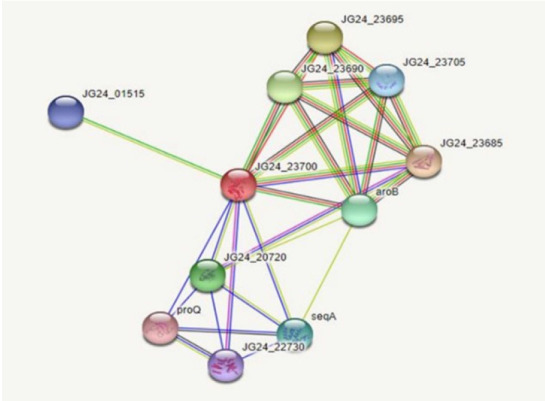
Proteins interacting with the main protein JG24_22730 at a specific site of DNA adenine methyltransferase and the functional partners.

Protein-protein interactions help decipher the interactome mechanisms of bacterial proteins. Neighboring proteins that scored greater than 0.7 were considered as high-confidence interacting proteins. The significant proteins identified were tryptophanyl t-RNA synthetase, ribulose phosphate 3-epiramase, DNA mismatch repair endonuclease MutH, three dihydro quinate synthase, and DamX (Fig. 3).

### Molecular docking

**Fig. 4.**
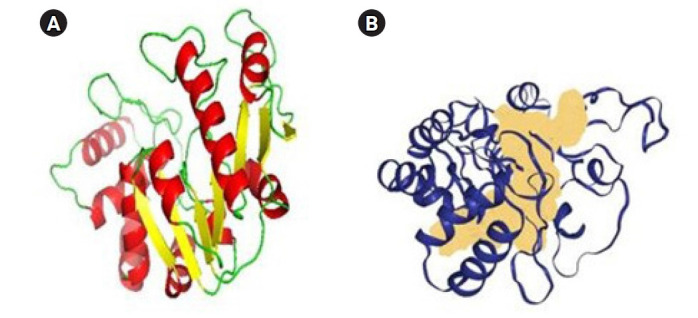
(A) Protein structure of A0A2U0NNR3 that was evaluated by using PyMOL. Red, helix; yellow, sheets; green, loops. (B) The active site is demonstrated by the region colored peach is the binding site of the protein predicted by DoGSiteScorer.

Initially, the protein structure A0A2U0NNR3 was searched in the PDB. The search results showed that the protein structure was not available. The protein sequence was submitted to SWISS-MODEL to construct the protein structure. The modeled structure was based on the retrieved template from the SWISS-MODEL template library of PDB ID: 4RTL. To ensure the structural quality of the model, the protein was first evaluated (Fig. 4). The evaluation was done using PROCHECK, ERRAT, and PRoSA. PROCHECK constructed a Ramachandran plot that gave 89.9% of residues in the favored regions, 9.4% in the additional allowed regions, and 0.4% in both the generously and disallowed regions. According to Kumar [51], a good-quality protein structure should have an overall percentage of residues in the favored and allowed regions of over 90%. Here, the model gives a total of 89.9% + 9.4% = 99.3%.

**Fig. 5.**
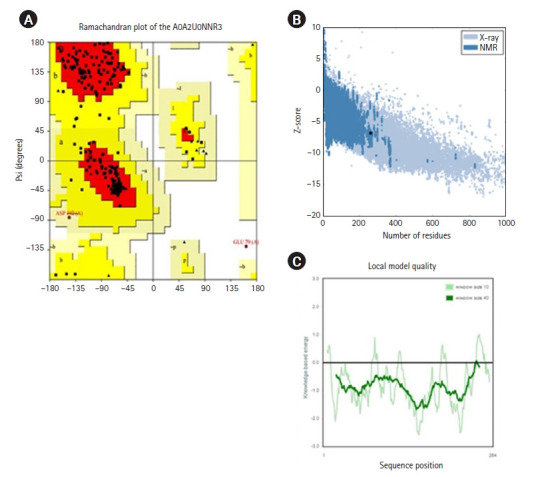
Structure quality assessment. (A) Ramachandran plot of the A0A2U0NNR3 protein by using SWISS-MODEL. (B) Z-score of the residues (ProSA). (C) Local model quality based on the sequence position. NMR, nuclear magnetic resonance.

The Z-score value from ProSA was –7.49, indicating that the model was made by the X-ray crystallography method. The ERRAT plot gave an overall factor quality score of 84.848, which was an average quality score. Based on the results, the protein model could be inferred for further study. The ligands that were sourced from the literature review were first analyzed for any violations of Lipinski rules (Fig. 5). Only ECGC was excluded as it gave two violations of the Lipinski rules: N or O>10, NH or OH>5.

The inhibitors were docked and defined to the predicted binding site with a dimension grid of 30 Å. Mahanine had the highest binding affinity score of –10.8 kcal/mol, followed by nanaomycin A (–10.0 kcal/mol), timethylaurintricarboxylic acid (–9.4 kcal/mol), and quercetin (–9.3 kcal/mol). Curcumin had the lowest binding affinity score of –8.5 kcal/mol. The molecular docking results are tabulated in Table 3.

### Virtual screening

Based on the binding affinity score, mahanine was further screened to identify a compound with a similar composition to that of mahanine. Mahanine is a carbazol alkaloid extracted from the plant species *Murraya koenigii* and has been previously reported as a DNA methyltransferases inhibitor. In order to identify new novel antibiotics derived from plants, a fingerprint search was performed. Based on the calculated fingerprint similarities, 22 compounds were found to be similar to mahanine. The names of three compounds were not available. Two compounds, koenimbine and grinibine, were reported to be carbazole alkaloids found in *M. koenigii* [52].

None of the ligands violated the Lipinski rules. The virtual screening was performed using AutoDock. The virtual screening results showed that koenimbine and claurailas B had the highest binding affinity score (–5.97 kcal/mol). Koenimbine is also one of the carbazole alkaloids of the same plant species and could be explored as a novel antibiotic (Table 3). Table 4 shows the ligand-binding score and drug-likeness property.

### ADMET test

ADMET testing was further conducted for koenimbine to understand the absorption, distribution, metabolism, excretion, and toxicity of the compound. The analysis was interpreted based on the guidelines of pkCSM [37]. Compound absorption analysis showed that the compound has high CaCO_2_ permeability. The water solubility was –4.618 log(mol/L). The compound absorbance in the human intestine was 93.479%, indicating a good absorption rate. Koenimbine was also demonstrated to be a P-glycoprotein II inhibitor, with significant implications for its pharmacokinetic effects.

In terms of distribution, koenimbine was seen to have a slightly low distribution in the tissue plasma, as the volume of distribution was 0.654 L/kg. The toxicity measurements using *Tetrahymena pyriformis* indicated that the compound was highly toxic against bacteria, with 0.948 μg/L. The compound was also seen to be mutagenic against bacteria indicating its ability to cause detrimental impacts on bacteria. The results are summarized in Table 5.

### Summary

We identified and prioritized a drug target (Dam) from 32 proteins of *K. pneumoniae* using subtractive genomics, based on drug properties, pocket analysis, pathway analysis, and structure analysis. Our proposed computational pipeline approach rapidly identified the drug target. Koenimbine, a natural bioactive chemical compound of *M. koenigii*, has the potential to be a novel antibiotic. Mahanine may also be a potential novel antibiotic for inhibiting Dam-containing bacterial pathogens.

## Figures and Tables

**Table 1.** Results of the ChEMBL approach for identified targets and DoGSiteScorer score pocket binding prediction of all three hypothetical proteins

UniProt ID	ChEMBL-targets identified	DoGSiteScorer
A0A3P4EC49	3	0.81
A0A3P4UG76	5	0.81
A0A2U0NNR3	10	0.82

**Table 2.** Virulence factors of the hypothetical protein A0A2U0NNR3

UniProt ID	Virulence factor
A0A3P5S6Q8	VFG010749 (sdhB) Dot/Icm type IV secretion system effector
	VFG001959 (hddC) capsular polysaccharide heptosyltransferase
	VFG000166 (pchE) dihydroaeruginoic acid synthetase PchE [Py]
	VFG000272 (ureE) urease accessory protein (ureE), metalloch
	VFG002139 (cdsD) type III secretion system inner membrane r
	

**Table 3.** Binding affinity between the A0A2U0NNR3 and the six ligands

Bioactive compound	Binding affinity (kcal/mol)
Curcumin	–8.5
Mahanine	–10.8
Nanaomycin A	–10.0
Parthenolide	–7.6
Quercetin	–9.3
Trimethylaurintricarboxylic acid	–9.4
	

**Table 4.** Binding affinity score between the A0A2U0NNR3 protein and 22 ligands based on similarity findings and drug-likeness properties

Natural product name	Product name	PubChem ID	Binding energy (kcal/mol)	Rotatable bonds	ADME TEST (Lipinski)
NPC162484	Koenimbine	97487	–5.97	2	Yes
NPC190007	Mahanimbilol	5353739	–5.95	2	Yes; 1 violation
NPC193777	Glybomine B	11208660	–5.96	2	Yes
NPC201697	Clausenawalline C	57409124	–5.96	2	Yes
NPC205934	Mahanimbine	167963	–5.95	2	Yes; 1 violation: MLOGP>4.15
NPC209769	Claurailas B	51039826	–5.97	2	Yes
NPC212535	3-Methyl-9H-Carbazol-2-Ol	3459141	–5.94	2	Yes
NPC243834	Sid506287	375146	–5.95	2	Yes
NPC267423	n.a.	375144	–5.96	2	Yes; 1 violation: MLOGP>4.15
NPC291535	Sid506290	375149	–5.94	2	Yes
NPC43787	Grinimibine	96943	–5.92	2	Yes
NPC470931	n.a.	71717470	–5.89	2	Yes; 1 violation: MLOGP>4.15
NPC475085	Sid506292	375151	–5.91	2	Yes
NPC475112	Sid506289	375148	–5.96	2	Yes
NPC476044	n.a.	44583761	–5.89	2	Yes; 1 violation: MLOGP>4.15
NPC476106	5-(3,5-Dimethyl-3,11-dihydropyrano[3,2-a]carbazol-3-yl)-2-methylpent-1-en-3-ol	375155	–5.96	2	Yes
NPC477532	3-[(2E)-3,7-dimethylocta-2,6-dienyl]-8-[3-[(2E)-3,7-dimethylocta-2,6-dienyl]-9-methoxy-3,5-dimethyl-11H-pyrano[3,2-a]carbazol-10-yl]-3,5-dimethyl-11H-pyrano[3,2-a]carbazol-7-ol	71725436	–5.95	2	No; 2 violations: MW>500, MLOGP>4.15
NPC48353	Glycoborinine	10446329	–5.95	2	Yes
NPC70259	(+)-Mahanimbicine	4072580	–5.94	2	Yes; 1 violation: MLOGP>4.15
NPC70956	Euchrestine B	15060943	–5.96	2	Yes; 1 violation: MLOGP>4.15
NPC72211	(-)-O-Methylmahanine	71716235	–5.96	2	Yes; 1 violation: MLOGP>4.15
NPC94943	Siamenol	477436	–5.95	2	Yes

**Table 5.** ADMET test results for koenimbine

	Value
Absorption	
Water solubility	–4.618
Numeric (log mol/L)	
CaCO_2_ permeabilty	1.411
Numeric (log Papp in 10^-6^ cm/s)	
Intestinal absorption (human) (% absorbed)	93.479
Skin permeability	–2.742
Numeric (log Kp)	
P-glycoprotein substrate	Yes
P-glycoprotein I inhibitor	No
P-glycoprotein II inhibitor	Yes
Distribution	
VDss (human)	0.654
Fraction unbound (human)	0.024
BBB permeability (log BB)	0.421
CNS permeability (log PS)	–1.789
Metabolism	
CYP2D6 substrate	No
CYP3A4 substrate	Yes
CYP1A2 inhibitor	Yes
CYP2C19 inhibitor	Yes
CYP2C9 inhibitor	Yes
CYP2D6 inhibitor	No
CYP3A4 inhibitor	No
Excretion	
Total clearance (log mL/min/kg)	0.477
Renal OCT2 substrate	No
Toxicity	
AMES toxicity	Yes
Max tolerated dose (human) (log mg/kg/day)	–0.312
Hepatotoxicity	Yes
Skin sensitization	No
Minnow toxicity (log mM)	–0.436

ADMET, absorption, distribution, metabolism, excretion, and toxicity; BBB, blood-brain barrier; CNS, central nervous system; OCT2, organic cation transporter 2.
